# Respiratory muscle dysfunction and exercise limitation in patients with moderate adolescent idiopathic scoliosis

**DOI:** 10.1186/1748-7161-7-S1-O62

**Published:** 2012-01-27

**Authors:** E Marco, JM Martínez-Llorens, SC Chiarella, MF Donaire, M Orozco-Levi, F Escalada

**Affiliations:** 1 Physical Medicine and Rehabilitation Dpt. Hospitales Mar-Esperança, Parc de Salut Mar. 2. Research Group, Injury, Immune Response and Pulmonary Function. IMIM Barcelona, Spain; 2 Pneumology Dpt. Hospital del Mar. Parc de Salut Mar; CIBERES (CIBER Respiratory Diseases, ISCIII). Barcelona, Spain; 3 Physical Medicine and Rehabilitation Dpt. Hospitals Mar-Esperança, Parc de Salut Mar. Barcelona, Spain; 4 Research Group, Injury, Immune Response and Pulmonary Function. IMIM.; Pneumology Dpt. Hospital del Mar. Parc de Salut Mar; CIBERES (CIBER Respiratory Diseases, ISCIII). Barcelona, Spain

## Background

Adolescent idiophatic scoliosis (AIS) can lead to ventilatory restriction, respiratory muscle weakness and exercise limitation. The aim of our study is to describe muscle weakness in AIS patients and its correlation with the curve magnitude.

## Materials and methods

Case-control study in with 85 patients with AIS and 25 healthy volunteers. AIS patients were classified into two groups determined by the curve mangitude): A (Cobb angle 25-40°) and B (Cobb angle >40°). Main outcomes were: respiratory muscle strength estimated by maximal inspiratory and expiratory preassures (MIP, MEP), peripherical muscle strength assessed in hands and lower limb muscles, respiratory function tests and exercise capacity. Statistical analysis: chi square test, t-Student and Pearson correlation coefficient.

## Results

The skeletal muscle function was decreased in AIS patients in comparison with the controls (p<0.001): MIP (%) 69.4 (SD 5.12) in Group A and 71 (SD 19) in Group B; in the control group MIP was 95 (SD 15); MEP (%) 63.2 (SD 17.9) and 69 (SD 19) for A and B respectively. In the control group was 91 (SD 18). We also decreased strength in lower limbs compared with the controls. There appeared to be no connection between spinal deformity and muscle function.

## Conclusions

The patients with AIS show a generalised muscle dysfunction, which contributes to the reduction in their exercise capacity, in absence of a correlation with the magnitude of spinal deformity.

